# CCND1 Stability Regulated by USP7 Deubiquitination Controls Glioma Radioresistance via Ferroptosis

**DOI:** 10.1155/ancp/1041908

**Published:** 2026-09-15

**Authors:** Yayu Zhuo, Chenrui Zhang, Xiaoyu Li, Zishuo Du, Yiren Rong, Di Zhang, Haijuan Mi, Xiaoyu Gu, Feng Wang, Haie Han, Jianliang Wu, Jianping Sun

**Affiliations:** ^1^ Department of Neurosurgery, The Second Hospital of Hebei Medical University, Shijiazhuang 050000, Hebei, China, hebmu.edu.cn; ^2^ Department of Thyroid and Breast Surgery, The Second Hospital of Hebei Medical University, Shijiazhuang 050000, Hebei, China, hebmu.edu.cn; ^3^ The Second Hospital of Hebei Medical University, Shijiazhuang 050000, Hebei, China, hebmu.edu.cn; ^4^ Shenze County People’s Hospital, Shijiazhuang 050000, Hebei, China; ^5^ Xingtang County Chinese Medicine Hospital, Shijiazhuang 050000, Hebei, China

**Keywords:** CCND1, ferroptosis, glioma, radioresistance, USP7

## Abstract

Dysregulation of Cyclin D1 (CCND1) is implicated in various cancers, but its specific contribution to glioma radioresistance and the associated regulatory mechanisms remain largely unexplored. This research aimed to elucidate the functional role and underlying mechanisms of CCND1 in glioma radioresistance. Our findings revealed that CCND1 is significantly upregulated in glioma tissues, which correlates with a poor prognosis. Furthermore, CCND1 knockdown inhibited proliferation, triggered apoptosis, and augmented radiosensitivity in glioma cells. Additionally, CCND1 depletion sensitized glioma cells to irradiation by driving ferroptosis, as indicated by elevated reactive oxygen species (ROS) production, lipid peroxidation, and intracellular Fe^2+^ accumulation. Mechanistically, USP7 was identified to stabilize the CCND1 protein via K48‐linked deubiquitination. Notably, the ectopic expression of USP7 reversed the impacts of CCND1 knockdown on radiosensitivity, ferroptosis, cellular proliferation, and apoptosis. Altogether, our data indicate that the stabilization of CCND1 mediated by USP7 promotes radioresistance in glioma by suppressing ferroptosis. Therefore, targeting USP7 may serve as a promising therapeutic approach to augment the efficacy of radiotherapy in glioma.

## 1. Introduction

At the most common primary malignancies in the brain, gliomas are characterized by their high aggressiveness and intrinsic resistance to standard treatments [1, 2]. Although substantial progress has been made in multimodal therapies involving surgical excision, chemotherapy, and radiation, the overall prognosis for glioma patients remains exceptionally poor [3, 4]. Specifically, radioresistance presents a major clinical obstacle, frequently resulting in local therapeutic failure, tumor relapse, and malignant advancement [5]. The inherent complexity of these tumors is additionally exacerbated by their pronounced intratumoral heterogeneity [6]. Notably, radiation tolerance in glioma is generally multifactorial, arising from complex cellular and molecular networks. These include modified DNA repair processes, altered cell cycle control, and the stimulation of prosurvival pathways [7]. Considering the indispensable role of radiation in managing glioma and the severe consequences of radioresistance, elucidating the underlying molecular mechanisms and discovering new therapeutic vulnerabilities are now pressing priorities in the field.

Cyclin D1 (CCND1), a crucial mediator of the G1/S phase transition during the cell cycle [8], is widely acknowledged as a key oncogene across multiple human cancers. Functioning as a fundamental subunit of the CCND1‐CDK4/6 complex, CCND1 drives the phosphorylation of the retinoblastoma protein, thereby facilitating cell cycle advancement and continuous proliferation [9, 10]. Abnormalities in CCND1 regulation are linked to the development of various cancers, frequently associating with aggressive disease features, unfavorable prognoses, and resistance to standard treatments [11–13]. Notably, elevated CCND1 levels in glioma samples are closely tied to poor patient survival and decreased sensitivity to radiation therapy [14]. Although elevated CCND1 is known to drive tumor cell multiplication in glioma [15], its exact contribution to radiation tolerance and the detailed molecular pathways involved remain largely unexplored.

USP7, also referred to as HAUSP, belongs to the ubiquitin‑specific protease family. It exerts deubiquitinase activity to stabilize a range of oncoproteins as well as tumor suppressors, thereby contributing critically to cancer pathogenesis [16]. Recent studies have demonstrated that USP7 is frequently overexpressed in various malignancies and promotes tumorigenesis through the deubiquitination and stabilization of key substrates. In melanoma, for example, USP7 physically associates with the PRC2 core subunit EZH2 and removes its polyubiquitin chains [17]. This deubiquitination event elevates H3K27 trimethylation levels and consequently represses the expression of FOXO1, a known tumor suppressor [17]. In triple‑negative breast cancer, USP7 stabilizes FOXM1, a master regulator of cell cycle and proliferation, thereby driving tumor growth independently of p53 [18]. Additionally, USP7 was reported to remove ubiquitin chains from PHF8, thereby enhancing its protein stability. PHF8, a histone demethylase, has been implicated in promoting breast carcinogenesis [19]. Furthermore, in gastric cancer, USP7 directly binds to and deubiquitinates PD‑L1, leading to its stabilization and facilitating immune evasion [20]. Collectively, these findings indicate that USP7 acts as an oncogenic deubiquitinase by controlling the stability of diverse proteins involved in cell cycle progression, epigenetic regulation, and immune checkpoint control. However, whether USP7 directly regulates CCND1 remains unknown.

Ferroptosis, a recently identified form of programmed cell death characterized by iron‐dependent lipid peroxidation, has attracted increasing attention within cancer research [21, 22]. This distinct pathway separates itself from traditional apoptosis, necrosis, and autophagy. Furthermore, it is involved in numerous physiological and disease states, exhibiting particular significance for tumor progression and treatment tolerance [23, 24]. Mounting evidence indicates that the susceptibility of tumor cells to ferroptosis could dictate their overall response to radiotherapy [21, 25]. Recent studies highlight that triggering this specific cell death pathway might serve as an effective approach to overcome radiation tolerance in gliomas, ultimately enhancing therapeutic efficacy [26, 27]. Nevertheless, the exact relationship between CCND1 expression and ferroptosis during glioma radioresistance remains largely uninvestigated.

In this present study, we seek to elucidate how CCND1 confers radiation tolerance in glioma cells. Our investigation primarily focuses on the functional consequences of CCND1 expression, emphasizing its effects on malignant phenotype and survival following irradiation. We also explore whether CCND1 depletion triggers ferroptosis and/or pyroptosis and investigate the upstream deubiquitinase that controls CCND1 stability. By mapping the molecular mechanisms linking CCND1 to glioma progression, this research highlights its potential as a viable therapeutic target. Ultimately, these insights may establish a robust basis for designing superior clinical strategies to enhance patient survival.

## 2. Materials and Methods

### 2.1. Cell Culture

Human glioma lines (U251 [RRID: CVCL_0021], U87 [RRID: CVCL_0022], A172 [RRID: CVCL_0033], HS683 [RRID: CVCL_0844], T98G [RRID: CVCL_0556], and LN229 [RRID: CVCL_A8V5]) and normal human astrocytes (SVGp12 [RRID: CVCL_3797]) were obtained from the Stem Cell Bank of the Chinese Academy of Sciences. U251, U87, A172, T98G, and LN229 cells were cultured in Dulbecco’s modified Eagle medium (DMEM, Thermo Fisher Scientific, USA). HS683 and SVGp12 cells were propagated in RPMI‐1640 medium (Thermo Fisher Scientific, USA) and Eagle’s minimum essential medium (EMEM, ATCC, USA), respectively. All culture media were supplemented with 10% fetal bovine serum (FBS) and 1% penicillin‐streptomycin (Thermo Fisher Scientific, USA). The cell cultures were maintained in a standard humidified incubator at 37°C with 5% CO_2_.

### 2.2. Lentiviral Construction and Cell Transduction

The CCND1‐targeting short hairpin RNA (shRNA) and USP7‐targeting shRNA were designed by Shanghai Yibeirui Bioscienceres, Co., Ltd. (Shanghai, China). Following reverse transcription into complementary DNA (cDNA), double‐stranded DNA fragments were generated and transformed into *Escherichia coli* (*E. coli*). Positive clones were subsequently verified via PCR. To produce lentiviral particles, the constructed plasmid packaging vectors were cotransfected into 293 T cells (RRID: CVCL_RU08), followed by viral concentration and purification. A nonrecombinant lentivirus served as the negative control. For USP7 overexpression, human USP7 cDNA was inserted into a vector supplied by the aforementioned vendor. U251 and U87 cells, seeded in six‐well plates, were transfected with the respective vectors using Lipofectamine 3000 (Thermo Fisher Scientific, USA).

### 2.3. Quantitative Real‐Time PCR (qPCR)

Total RNA was extracted from the cellular cells using TRIzol reagent (Thermo Fisher Scientific, USA) following the manufacturer’s instructions. RNA yield and purity were evaluated via a NanoDrop spectrophotometer (Thermo Fisher Scientific, USA). Subsequently, 1 μg of total RNA was reverse‐transcribed into cDNA with a cDNA synthesis kit (Bio‐Rad, USA). Real‐time qPCR was performed on a StepOnePlus system using SYBR Green PCR Master Mix (Applied Biosystems, USA). The thermocycling conditions included an initial denaturation at 95°C for 10 min, followed by 40 cycles of 95°C for 15 s, a primer‐specific annealing temperature for 30 s, and 72°C for 30 s. Amplification specificity was confirmed via melting curve analysis. Relative mRNA levels were calculated utilizing the 2^−ΔΔCt^ approach, normalizing to GAPDH as the internal control.

### 2.4. Western Blot

Protein samples were separated via 10% SDS‐PAGE and subsequently transferred to nitrocellulose membranes (Bio‐Rad, USA). Nonspecific binding was blocked by incubating the membranes in 5% nonfat milk prepared in TBST for 1 h at room temperature. The blots were then probed for 2 h with the following primary antibodies: CCND1, USP7, pro‐caspase‐1, cleaved caspase‐1, GSDMD, cleaved N‐terminal GSDMD, IL‐18, IL‐1β, and GAPDH (RRID: AB_3102089, MedChemExpress). Following standard TBST washes, the membranes were incubated with HRP‐conjugated secondary antibodies for an additional 1 h. Protein bands were visualized utilizing an enhanced chemiluminescence (ECL) kit (PerkinElmer, USA), and images were acquired using the ChemiDoc MP Imaging System (Bio‐Rad, USA).

### 2.5. Cell Counting Kit‐8 (CCK‐8) Assay

Cell viability was assessed using the CCK‐8 assay (Dojindo, Japan). Cells were seeded into 96‐well plates at a density of 5000 cells/well in 100 μL of complete medium and cultured at 37°C in a humidified 5% CO_2_ atmosphere. At 24, 48, and 72 h posttreatment, 10 μL of CCK‐8 reagent was added to each well, followed by incubation for an additional 2 h. The optical density (OD) at 450 nm was measured using a Varioskan LUX microplate reader (RRID: SCR_014350, Varioskan LUX, Thermo Fisher Scientific). Cell viability percentages were calculated by normalizing the absorbance of each experimental group to its initial baseline value at 0 h.

### 2.6. Colony Formation Assay

For the colony formation assay, harvested cells were resuspended in DMEM and seeded into six‐well plates at 500 cells/well. The cultures were maintained in a 37°C, 5% CO_2_ incubator for 2 weeks, with the medium replaced every 3 days. Following this incubation period, the colonies were fixed using 4% paraformaldehyde (Electron Microscopy Sciences, USA) for 20 min at ambient temperature. Subsequently, the plates were stained with 0.1% crystal violet (Sigma–Aldrich, USA) for 30 min. After rinsing with phosphate‐buffered saline (PBS) (Sigma–Aldrich, USA) and air‐dried, colonies comprising more than 50 cells were manually counted under an Olympus light microscope (Japan) to evaluate plating efficiency.

### 2.7. Flow Cytometry

Cells were trypsinized, washed twice with cold PBS, and subsequently resuspended in 1× binding buffer at a concentration of 1 × 10^6^ cells/mL. A 100‐μL aliquot of this cell suspension was transferred into a flow cytometry tube, followed by the addition of 5 μL of Annexin V‐FITC and 5 μL of PI. After gentle mixing, the samples were incubated in the dark for 15 min at room temperature. Following incubation, 400 μL of 1× binding buffer was added to each tube. Flow cytometry data acquisition was performed within 1 h using a BD FACSCanto II flow cytometer (RRID: SCR_014527, BD FACSCanto II, BD Biosciences, USA), and the resulting data were evaluated via FlowJo software (RRID: SCR_008520, Tree Star, USA).

### 2.8. Animal Experiments

Male BALB/c nude mice (6–8 weeks old, body weight 20–25 g, *n* = 12) were obtained from Charles River Laboratories. The animals were randomly divided into two groups: the control group (sh‐NC) and the treatment group (sh‐CCND1). A cell suspension (0.2 mL) containing 2 × 10^4^ cells was prepared in a mixture of culture medium (DMEM supplemented with 10% FBS) and Matrigel at a 1:1 ratio and then injected subcutaneously into the left flank of each mouse. Both research staff and veterinary personnel monitored the mice twice daily. Tumor growth was evaluated three times per week, with tumor dimensions recorded weekly using digital calipers. Tumor volume (mm^3^) was calculated as (width × height^2^)/2. After a 28‐day observation period, all animals were anesthetized by inhaling 3% isoflurane vapor through a mask. All procedures involving animals were carried out in compliance with institutional guidelines and received approval from the Institutional Animal Care and Use Committee of The Second Hospital of Hebei Medical University.

### 2.9. Radiation Sensitivity Assay

Cells were seeded into six‐well plates at a density of 500 cells/well and allowed to attach for 24 h. The cultures were then irradiated with X‐rays at escalating doses (0–8 Gy) utilizing a PRIMUS linear accelerator (Siemens Healthcare, USA). Detailed irradiation conditions were established in accordance with a previous protocol [28]. After exposure, the plates were incubated for another 14 days to facilitate colony formation. Macroscopic colonies consisting of more than 50 cells were scored as surviving clonogens.

### 2.10. Reactive Oxygen Species (ROS) Detection

Intracellular ROS levels were assessed using the fluorescent probe DCFH‐DA, provided in a ROS detection kit (Beyotime, China). Cells were incubated with 5 μM DCFH‐DA for 30 min at 37°C in the dark. After the incubation, cells were collected, and fluorescence intensity was measured with a spectrofluorometer at excitation and an emission wavelength of 485 and 535 nm, respectively.

### 2.11. Iron Assay

Intracellular ferrous iron (Fe^2+^) levels were measured using a colorimetric assay kit (Elabscience, Wuhan, China) according to the manufacturer’s protocol. Cell supernatants were collected from U251 and U87 cells after experimental treatments. An aliquot of 75 μL from each sample was mixed with 300 μL of iron chromogenic reagent, and the mixture was centrifuged at 3000 × g for 10 min at 4°C. The Fe^2+^ concentrations were determined by calculating sample values based on the OD absorbance readings at 530 nm.

### 2.12. Quantification of Intracellular Glutathione (GSH) Levels

Cellular GSH levels were measured using a commercially available colorimetric microplate assay kit (Beyotime, China) following the supplier’s instructions. Aliquots of 50 μL from each sample and standard were dispensed into a 96‐well microplate. A freshly prepared mixture containing 5,5′‐dithiobis‐(2‐nitrobenzoic acid) (DTNB) and GSH reductase, referred to as the working solution, was added to each well. The reaction was initiated by the addition of NADPH solution, and the plate was incubated at 25°C for 25 min in the dark. Subsequently, absorbance was recorded at 450 nm utilizing a Synergy H1 microplate reader (RRID: SCR_014332, BioTek Instruments, Winooski, USA). GSH concentrations were calculated from a standard curve and normalized to total protein content.

### 2.13. Enzyme‐Linked Immunosorbent Assay (ELISA)

The concentration of 4‐HNE, a marker of lipid peroxidation, was measured in cellular samples using a universal 4‐HNE ELISA kit (FineTest, Wuhan, China) according to the manufacturer’s protocol. Levels of malondialdehyde (MDA) in cell culture supernatants were assessed with commercial ELISA kits (Esebio, Shanghai, China), following the instructions provided by the manufacturer. Lactate dehydrogenase (LDH) release was detected using LDH Cytotoxicity Assay Kit (Beyotime).

### 2.14. Coimmunoprecipitation (Co‐IP) Assay

Protein extraction started with cell lysis in a buffer containing 50 mM Tris‐HCl (pH 7.4), 150 mM NaCl, 1 mM EDTA, 1% NP‐40, and protease inhibitors. The lysates were then centrifuged to remove insoluble material. The supernatants were collected and precleared with protein A/G agarose beads (RRID: SCR_018616, Abcam, UK) at 4°C for 2 h, followed by centrifugation at 1000 g for 5 min. Subsequently, the cleared lysates were incubated with specific antibodies overnight at 4°C with gentle agitation. Immunoprecipitation was carried out by adding protein A/G agarose beads for an additional 2 h. After extensive washing with lysis buffer, the beads were resuspended in 2 × Laemmli buffer and heated at 100°C for 10 min, and the eluates were analyzed by Western blotting.

### 2.15. Immunofluorescence Colocalization

Cells grown on coverslips were fixed with 4% paraformaldehyde (EMS, USA) and permeabilization with 0.1% Triton X‐100 (Sigma–Aldrich, USA). After blocking with 5% BSA, the coverslips were incubated with primary antibodies overnight at 4°C, followed by Alexa Fluor–conjugated secondary antibodies (Thermo Fisher Scientific, USA) for 1 h at room temperature. Nuclei were counterstained with DAPI (RRID: SCR_018636, Thermo Fisher Scientific, USA). The coverslips were mounted using ProLong Gold (RRID: SCR_015961, Thermo Fisher Scientific, USA), and images were acquired on a Nikon Eclipse Ti confocal microscope (RRID: SCR_016402).

### 2.16. Ubiquitination Assay

Cells were cotransfected with plasmids encoding CCND1‐Flag, USP7‐Myc, and various HA‐tagged ubiquitin forms (wild type, K48R, and K63R) using Lipofectamine 3000 (Thermo Fisher Scientific, USA) following the manufacturer’s instructions. For immunoprecipitation, 500 μg of total protein was incubated with anti‐Flag agarose beads (NuoyiBio, China) at 4°C overnight. The immunoprecipitated complexes and total cell lysates were resolved on 10% SDS‐PAGE and transferred onto PVDF membranes (Millipore, USA). The membranes were blocked with 5% nonfat milk in TBST for 60 min at room temperature and then incubated with primary antibodies overnight at 4°C. The antibodies used were anti‐HA (Sigma–Aldrich, USA), anti‐Myc (Sigma–Aldrich), and anti‐Flag (Sigma–Aldrich). After washing, the membranes were developed by chemiluminescence, following standard Western blot procedures. Ubiquitination of CCND1 was evaluated by detecting HA‐tagged ubiquitin in the immunoprecipitated samples, while protein expression levels were determined from total cell lysates.

### 2.17. Statistical Analyses

All independent experiments were carried out in triplicate, with the results presented as the mean ± standard deviation (SD). Statistical comparisons were conducted using Student’s *t*‐test, where a *p*‐value of  < 0.05 was considered to be statistically significant.

## 3. Results

### 3.1. CCND1 Exhibits Aberrant Expression in Glioma and Correlates With Poor Clinical Outcomes

**Figure 1:**
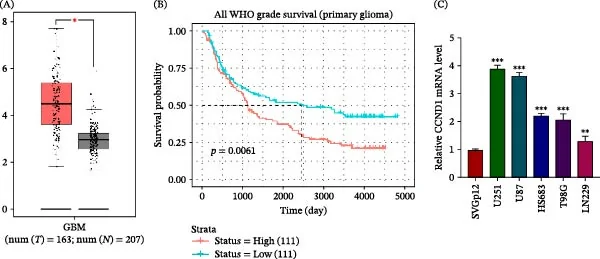
CCND1 expression in glioma and its clinical significance. (A) Comparative analysis of CCND1 expression levels in glioma tissues versus normal tissues, as sourced from The Cancer Genome Atlas (TCGA) database. (B) Further analysis of TCGA database indicates a negative correlation between CCND1 expression levels and the prognosis of patients with WHO grade II glioma. (C) Comparative expression profiling of CCND1 in glioma cell lines versus normal cell lines. Experiments were performed in triplicate.  ^∗∗^ *p* < 0.01,  ^∗∗∗^ *p* < 0.001 vs. SVGp12.

To assess the clinical relevance of CCND1 in glioma, we first examined its expression levels using The Cancer Genome Atlas (TCGA) database. Our results showed that CCND1 was significantly upregulated in glioma tissues relative to normal brain tissues (Figure 1A). Based on this distinct expression pattern, we further evaluated its prognostic value through survival analysis. Notably, Kaplan–Meier analysis indicated that glioma patients with elevated CCND1 expression had significantly shorter overall survival than those with low expression (Figure 1B), suggesting that CCND1 may serve as a potential prognostic marker. To validate these clinical observations at the cellular level, we measured CCND1 expression across a panel of glioma cell lines, using the normal human astrocyte cell line SVGp12 as a reference. Consistent with our bioinformatic analysis, CCND1 was markedly upregulated across all tested glioma cell lines, with particularly pronounced expression levels observed in U251 and U87 cells (Figure 1C). Collectively, these results indicate that CCND1 holds potential as both a prognostic biomarker and a therapeutic target in glioma.

### 3.2. CCND1 Silencing Suppresses Glioma Cell Proliferation and Promotes Apoptosis

To explore the functional role of CCND1 in glioma progression, we generated CCND1‐knockdown models in U251 and U87 cells using shRNA. The silencing efficiency was first validated at both the transcriptional and protein levels. RT‐qPCR analysis revealed a pronounced decrease in CCND1 mRNA expression following shRNA transfection (Figure 2A), which was supported by Western blot data showing a corresponding reduction in CCND1 protein levels (Figure 2B). The biological effects of CCND1 depletion were then systematically assessed using multiple assays. CCK‐8 assays showed that CCND1 silencing significantly impaired cell viability in both glioma cell lines relative to their respective controls (Figure 2C). This antiproliferative effect was further confirmed by colony formation assays, where CCND1‐depleted cells exhibited a marked decrease in colony‐forming ability (Figure 2D), indicating an essential role of CCND1 in sustaining the long‐term proliferative capacity of glioma cells. Given the strong impact on cell viability, we next investigated whether CCND1 knockdown influences cell survival through flow cytometry analysis. Notably, both U251 and U87 cells transduced with sh‐CCND1 displayed significantly higher apoptotic fractions compared to control cells (Figure 2E). In mouse xenograft models, tumors derived from CCND1‐knockdown cells exhibited significantly reduced volume and weight (Figure 2F). These in vivo findings indicate that CCND1 silencing inhibits glioma progression. Collectively, these findings demonstrate that CCND1 acts as a key regulator of glioma cell survival by promoting proliferation and suppressing apoptosis.

Figure 2Effects of CCND1 silencing on glioma cells. (A) RT‐qPCR was used to verify the reduction of CCND1 mRNA levels following shRNA‐induced knockdown in glioma cells. (B) Western blotting confirmed the decrease in CCND1 protein levels following successful gene silencing. (C) Cell viability was determined using the CCK‐8 assay following CCND1 knockdown in U251 and U87 cell lines. (D) The colony formation assay evaluated the colony‐forming capacity following CCND1 silencing. (E) Flow cytometry quantified apoptosis in cells post‐CCND1 knockdown. (F) The volume and weight of xenograft tumors were measured following CCND1 silencing. Experiments were performed in triplicate.  ^∗∗∗^ *p* < 0.001 vs. sh‐NC.

**Figure:**
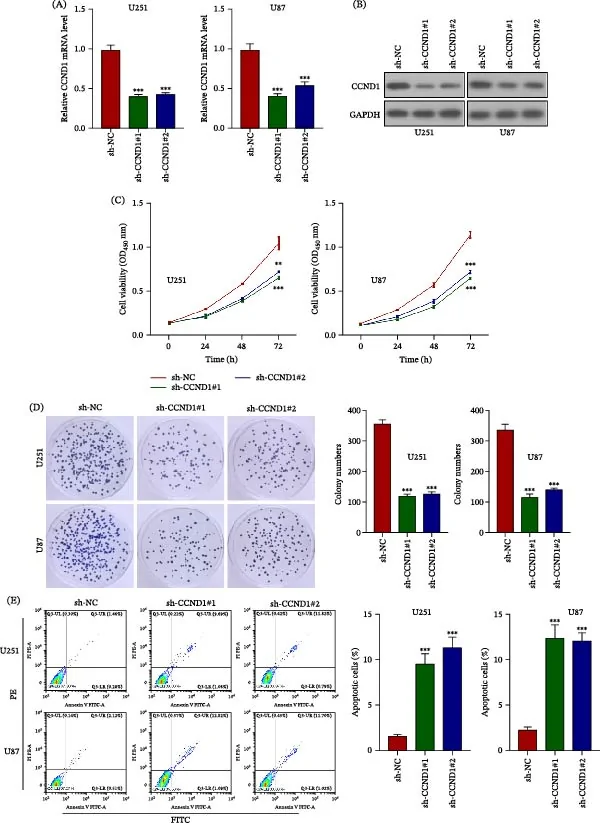

**Figure:**
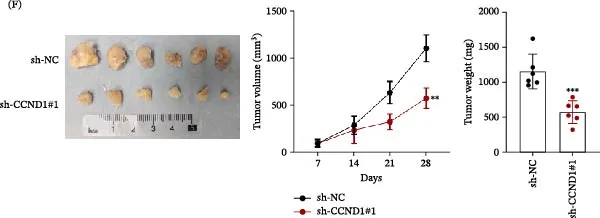

### 3.3. CCND1 Depletion Sensitizes Glioma Cells to Radiotherapy

**Figure 3:**
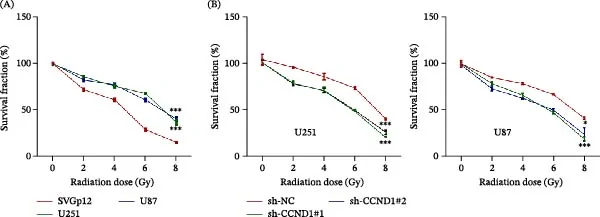
CCND1 depletion enhances radiotherapy sensitivity in glioma cells. (A) Comparative analysis of radiation sensitivity between normal astrocytes (SVGp12) and glioma cell lines (U251, U87) across varying radiation doses.  ^∗∗∗^ *p* < 0.001 vs. SVGp12. (B) Survival fraction analysis under radiation exposure indicates a marked enhancement in radiosensitivity of glioma cells upon CCND1 depletion.  ^∗^ *p* < 0.05,  ^∗∗∗^ *p* < 0.001 vs. sh‐NC. Experiments were performed in triplicate.

To explore the relationship between CCND1 expression and radiotherapy resistance in glioma, we first compared radiation sensitivity among normal astrocytes (SVGp12) and glioma cell lines (U251 and U87). Cells were exposed to increasing doses of radiation (0–8 Gy), and their survival fractions were measured. Intriguingly, both U251 and U87 cells showed significantly higher survival fractions than SVGp12 cells across all radiation doses, suggesting an inherent radioresistance in glioma cells (Figure 3A). To further examine whether CCND1 contributes to this resistance, we subjected our CCND1‐knockdown glioma cells to radiation treatment. Notably, CCND1‐depleted glioma cells displayed markedly enhanced radiosensitivity, as reflected by substantially reduced survival fractions relative to their control cells (Figure 3B), revealing a critical role of CCND1 in maintaining radioresistance in glioma.

### 3.4. CCND1 Depletion Enhances Radiosensitivity Through Ferroptosis Activation in Glioma Cells

**Figure 4:**
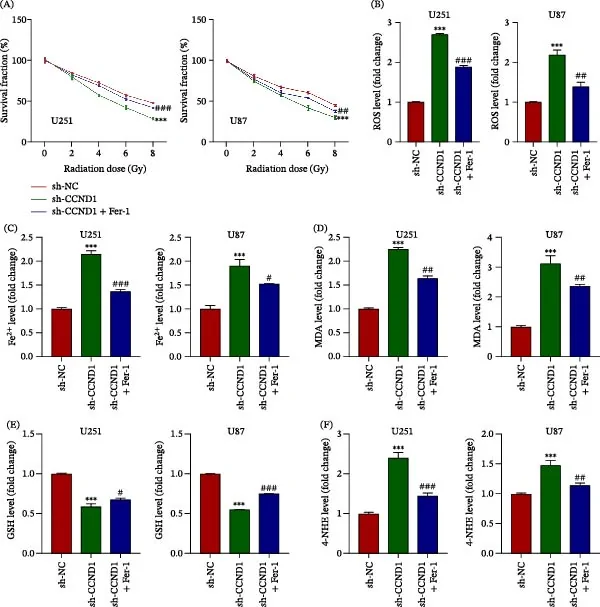
CCND1 depletion enhances radiosensitivity through ferroptosis in glioma cells. (A) The radiosensitivity of CCND1‐depleted U251 and U87 cells was assessed in the presence of the ferroptosis inhibitor ferrostatin‐1. (B) Cellular ROS levels were measured following CCND1 silencing and ferrostatin‐1 treatment. (C) Intracellular Fe2+ levels were quantified in CCND1‐depleted cells with and without ferrostatin‐1. (D, E) MDA and 4‐HNE levels, indicators of ferroptosis, were measured in CCND1‐depleted cells and compared with ferrostatin‐1–treated cells. (F) GSH levels were analyzed in CCND1‐depleted cells and ferrostatin‐1 administration. Experiments were performed in triplicate.  ^∗∗∗^ *p* < 0.001 vs. sh‐NC; ^#^ *p* < 0.05, ^*##*^ *p* < 0.01, and ^*###*^ *p* < 0.001 vs. sh‐CCND1+Fer‐1.

To explore the mechanistic connection between CCND1 and radioresistance, we examined whether ferroptosis, a regulated cell death process driven by iron‐dependent lipid peroxidation, might be involved. We first asked if inhibiting ferroptosis could affect the radiosensitization induced by CCND1 knockdown. Notably, treatment with ferrostatin‐1 (Fer‐1), a selective ferroptosis inhibitor, markedly reversed the enhanced radiosensitivity seen in CCND1‐depleted U251 and U87 cells (Figure 4A), pointing to a key role of ferroptosis in this response. We then systematically assessed several ferroptosis‐related parameters. CCND1 silencing led to a marked rise in cellular ROS levels, indicative of increased lipid peroxidation, and this effect was effectively blunted by Fer‐1 treatment (Figure 4B). In line with ferroptosis activation, CCND1‐depleted cells displayed higher intracellular Fe^2+^ levels (Figure 4C). Moreover, we observed significant elevations in classical ferroptosis markers, including MDA and 4‐HNE (Figure 4D,E), along with a concurrent reduction in GSH content (Figure 4F). Importantly, all of these ferroptotic changes were effectively reversed by Fer‐1 administration. In addition to ferroptosis, we explored whether pyroptosis, another form of regulated cell death, might also be activated. As shown in Supporting Information 1: Figure S1A–C, CCND1 knockdown led to increased GSDMD‐N, cleaved caspase‐1, IL‐1β, IL‐18, and LDH release, which were reversed by the pyroptosis inhibitors Z‐YVAD‐FMK and DSF, indicating pyroptosis activation (Supporting Information 1: Figure S1A–C). Collectively, these findings strongly suggested that CCND1 knockdown enhances radiotherapy sensitivity in glioma cells by activating both ferroptosis and pyroptosis, indicating a multifaceted cell death response.

### 3.5. USP7 Specifically Mediates K48‐Linked Deubiquitination of CCND1

**Figure 5:**
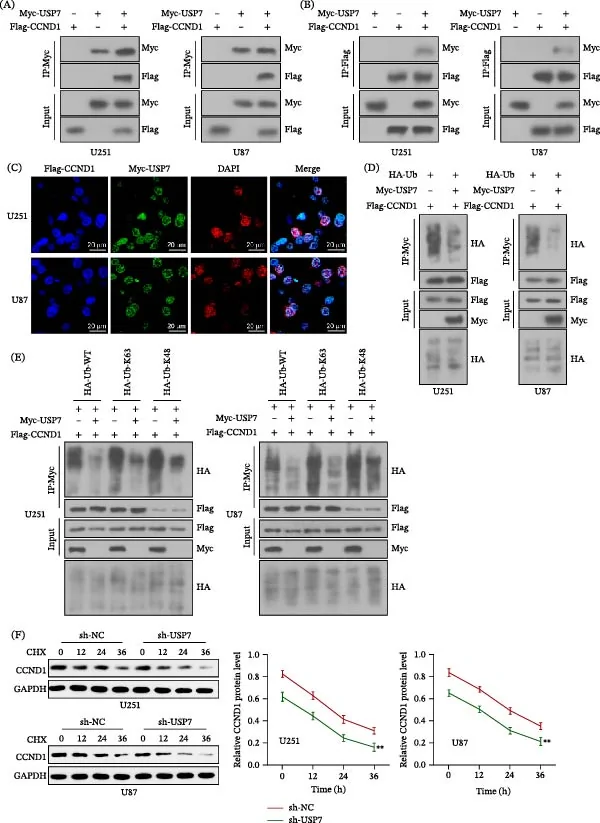
USP7‐mediated deubiquitination of ccnd1 in glioma cells. (A, B) Coimmunoprecipitation assays substantiated the binding between CCND1 and USP7 within glioma cell lines. (C) Immunofluorescence analysis showed the colocalization of CCND1 and USP7 in the nuclei of glioma cells. Scale bar, 20 μm. (D) Ubiquitination of CCND1 is diminished by the overexpression of USP7 in U251 and U87 cells. (E) Overexpression of USP7 preferentially reduced K48‐linked ubiquitination of CCND1 while leaving K63‐linked ubiquitination largely unaffected. Cells were transfected with HA‐Ub‐WT, HA‐Ub‐K48, or HA‐Ub‐K63 as indicated. (F) Western blot and statistical analysis of CCND1 in USP7 in U251 cells which transfected with sh‐NC or sh‐USP7 and treated with CHX. Experiments were performed in triplicate.  ^∗∗^ *p* < 0.01 vs. sh‐NC.

To explore the molecular mechanisms underlying CCND1 regulation, we examined potential protein–protein interactions that could influence CCND1 stability. Using reciprocal co‐IP assays, we identified a strong physical interaction between CCND1 and USP7, a well‐characterized deubiquitinating enzyme (Figure 5A,B). This interaction was further confirmed by immunofluorescence analysis, which showed clear colocalization of CCND1 and USP7 in glioma cells (Figure 5C). Given the known role of USP7 in protein deubiquitination, we next assessed its effect on CCND1 ubiquitination status. Ectopic expression of USP7 in both U251 and U87 cells resulted in a marked decrease in CCND1 ubiquitination levels (Figure 5D). To further determine the specificity of USP7‐mediated deubiquitination, we employed HA‐tagged ubiquitin mutants that specifically target K48 or K63 linkages. Notably, USP7 overexpression preferentially reduced K48‐linked ubiquitination of CCND1, whereas it had no significant impact on K63‐linked ubiquitination (Figure 5E). To directly evaluate the effect of USP7 on CCND1 protein stability, we first confirmed the knockdown efficiency of sh‐USP7 in U251 and U87 cells, as shown in Supporting Information 1: Figure S1D. Cycloheximide (CHX) chase assays were then performed. Cells were transfected with sh‐NC or sh‐USP7 and subsequently treated with CHX for the indicated times (0, 12, 24, and 36 h). As shown in Figure 5F, USP7 knockdown markedly accelerated the degradation of endogenous CCND1 compared with the control group. In further support of this, ectopic overexpression of USP7 prolonged the half‐life of CCND1 protein in U251 cells (Supporting Information 2: Figure S2A). Moreover, to ascertain whether the downregulation of CCND1 induced by USP7 depletion is dependent on the proteasome pathway, U251 cells were treated with the specific proteasome inhibitor MG132. Notably, MG132 treatment successfully restored the reduction of CCND1 protein levels caused by USP7 silencing (Supporting Information 2: Figure S2B). These findings uncover a regulatory mechanism by which USP7 specifically removes K48‐linked ubiquitin chains from CCND1, thereby preventing its proteasomal degradation and preserving its stability in glioma cells.

### 3.6. USP7 Overexpression Counteracts CCND1 Silencing–Induced Radiosensitization and Ferroptosis

**Figure 6:**
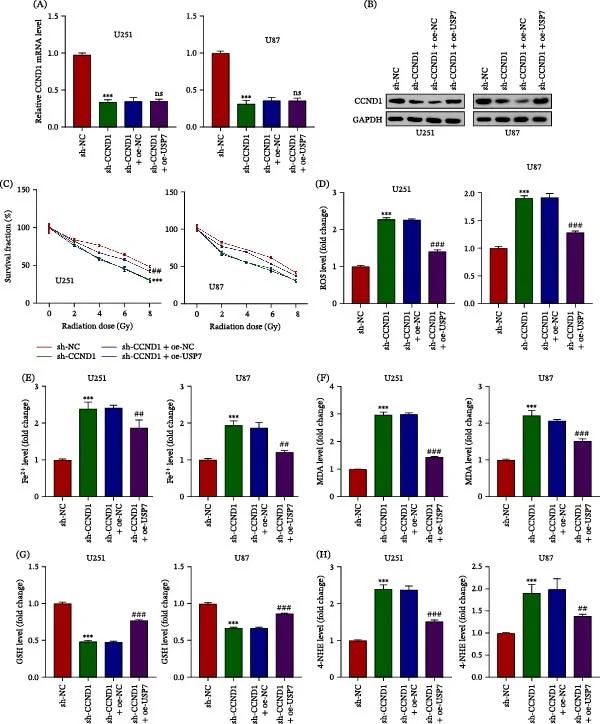
USP7 counteracted radiosensitization and ferroptosis induced by CCND1 silencing. (A) RT‐qPCR analysis of CCND1 mRNA levels in U251 and U87 cells cotransfected with sh‐CCND1 and oe‐USP7. (B) Western blot analysis demonstrating the restoration of CCND1 protein levels in cells cotransfected with sh‐CCND1 and oe‐USP7. (C) The radiosensitizing effect of CCND1 knockdown was assessed in the presence of USP7 overexpression. (D–F) USP7 overexpression was shown to reverse ferroptotic changes in CCND1‐depleted cells, including decreased ROS levels and intracellular Fe^2+^ accumulation. (G, H) Ferroptosis‐associated parameters induced by CCND1 silencing, such as lipid peroxidation products and GSH content, were restored by USP7 overexpression. Experiments were performed in triplicate.  ^∗∗∗^ *p*  < 0.001 vs. sh‐NC; ^*##*^ *p* < 0.01, ^*###*^ *p* < 0.001 vs. sh‐CCND1+oe‐USP7. ns, no significance vs. sh‐CCND1+oe‐NC.

To further confirm the functional relationship between USP7 and CCND1 in glioma radioresistance, we established USP7‐overexpressing cell models in both U251 and U87 cell lines. Successful USP7 overexpression was first verified at both the transcriptional and protein levels (Supporting Information 2: Figure S2C,D). Importantly, as USP7 regulates CCND1 via posttranslational deubiquitination, we investigated whether USP7 overexpression could specifically rescue CCND1 protein levels in sh‐CCND1–transfected cells. As expected, RT‐qPCR analysis revealed that USP7 overexpression did not rescue the reduced CCND1 mRNA levels induced by sh‐CCND1 (Figure 6A). However, Western blot analysis demonstrated that concurrent overexpression of USP7 significantly restored CCND1 protein levels compared to the sh‐CCND1 group (Figure 6B). This stark contrast between mRNA and protein levels unequivocally indicates that USP7 exerts its rescue effect by stabilizing the residual CCND1 protein that escapes shRNA‐mediated degradation. Notably, USP7 overexpression significantly diminished the radiosensitizing effects induced by CCND1 knockdown, as evidenced by increased survival fractions following radiation exposure in both glioma cell lines (Figure 6C). This observation indicates that USP7‐mediated CCND1 stabilization is critical for maintaining radioresistance in glioma cells. We next asked whether USP7 modulates CCND1‐dependent ferroptosis regulation. Remarkably, USP7 overexpression effectively reversed the ferroptotic phenotypes observed in CCND1‐depleted cells, including elevated ROS production and increased intracellular Fe^2+^ accumulation (Figure 6D–F). Moreover, the changes in ferroptosis‐associated parameters induced by CCND1 silencing, including elevated levels of lipid peroxidation products (4‐HNE and MDA) and reduced GSH content, were significantly restored by USP7 overexpression (Figure 6G,H). Collectively, these findings suggest that USP7 overexpression counteracted the effects of CCND1 silencing on radioresistance and ferroptotic processes.

### 3.7. USP7 Overexpression Abrogates the Effects of CCND1 Silencing on the Proliferation and Apoptosis

**Figure 7:**
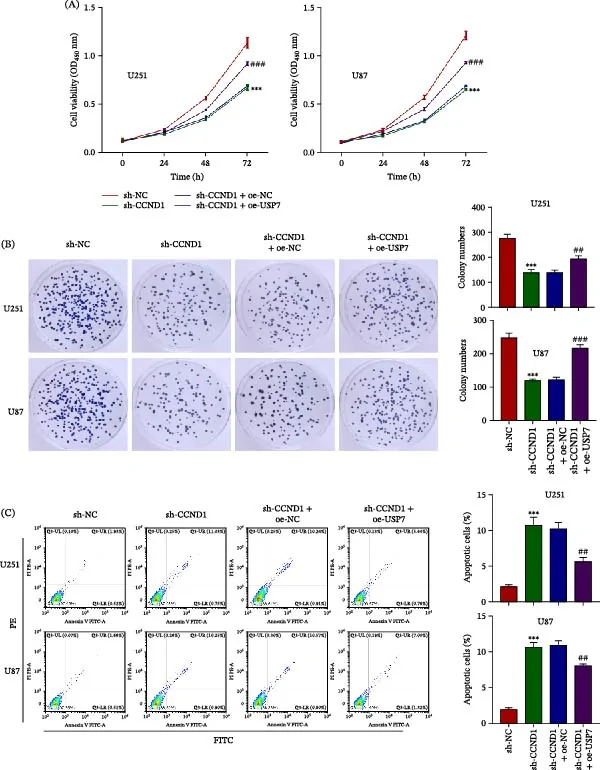
USP7 overexpression restored cellular functions suppressed by CCND1 silencing. (A) The cell viability in CCND1‐knockdown cells was assessed following USP7 overexpression in U251, and U87 cells were evaluated by CCK8. (B) The colony‐forming ability in CCND1‐knockdown cells was assessed following USP7 overexpression. (C) The induction of apoptosis by CCND1 silencing was analyzed in the presence of USP7 overexpression. Experiments were performed in triplicate.  ^∗∗∗^ *p* < 0.001 vs. sh‐NC; ^*##*^ *p* < 0.01, ^*###*^ *p* < 0.001 vs. sh‐CCND1+oe‐USP7.

To further explore the functional interplay between USP7 and CCND1 in glioma cell survival, we examined whether USP7 overexpression could counteract the cellular effects induced by CCND1 silencing. Consistent with our earlier observations, CCND1 knockdown significantly impaired cell viability in both U251 and U87 cells. Importantly, concurrent USP7 overexpression substantially restored cell viability in CCND1‐depleted cells (Figure 7A), suggesting a protective role of USP7 against CCND1 deficiency. The rescue effect of USP7 was further confirmed through colony formation assays. While CCND1 silencing markedly reduced the colony‐forming capacity of glioma cells, USP7 overexpression significantly recovered both colony numbers and sizes in CCND1‐knockdown cells (Figure 7B), indicating that USP7 can effectively compensate for CCND1 deficiency in maintaining long‐term proliferative potential. We next evaluated the impact of USP7 overexpression on apoptosis induced by CCND1 silencing. Flow cytometry analysis revealed that the increased apoptotic cell populations observed in CCND1‐depleted cells were significantly diminished upon USP7 overexpression (Figure 7C). Collectively, these results demonstrate that USP7 overexpression significantly abrogated the detrimental effects of CCND1 silencing on cell proliferation and survival.

## 4. Discussion

In this study, we observed that CCND1 was aberrantly overexpressed in gliomas, and this upregulation correlated with poor patient prognosis. Knockdown of CCND1 inhibited cell proliferation and promoted apoptosis in glioma cells. Moreover, CCND1 suppression not only increased the radiosensitivity of glioma cells but also induced ferroptosis. These findings highlight the potential therapeutic significance of targeting CCND1 in glioma management.

CCND1 has been extensively studied in various cancer types, including breast, colorectal, and bladder cancers, consistently linking its amplification to tumor progression [29–31]. Studies in gliomas have also reported elevated CCND1 levels, correlating with aggressive tumor characteristics and poor prognosis [32]. Our observations align with this body of evidence, showing significant upregulation of CCND1 in gliomas that negatively correlates with patient survival, highlighting its potential utility as a prognostic biomarker for glioma. The essential role of CCND1 in positively regulating malignant phenotypes of cancer cells, including proliferation, apoptosis resistance, and therapeutic tolerance, has been well documented across various cancer types [11–13]. Notably, our results showed that CCND1 knockdown reduces cell viability and proliferation while markedly enhancing apoptosis in glioma cells, indicating that CCND1 is a key factor in sustaining glioma cell survival.

Radioresistance represents a major obstacle contributing to treatment failure and poor prognosis in cancer patients. Tumor cell heterogeneity, arising from genetic, transcriptomic, epigenetic, and/or phenotypic variations, leads to differential responses to therapy [33]. Irradiation‐induced damage disrupts the cell cycle and impairs the self‐repair capacity of tumor cells, thereby promoting resistance to radiotherapy [34]. Furthermore, tumors may develop adaptive responses to low‐dose radiation, becoming highly resistant and more aggressive, which has spurred considerable in strategies to enhance radiosensitivity [35]. Notably, CCND1 expression levels have been positively associated with chemotherapy and radiotherapy resistance in low‐grade gliomas [36]. Our results showed that CCND1 knockdown enhances radiosensitivity, with cell viability further declining as radiation doses increase, suggesting that targeting CCND1 may represent a promising strategy to overcome radioresistance in glioma treatment.

Ferroptosis is a regulated cell death process driven by iron‐dependent lipid peroxidation and has shown considerable potential in suppressing tumor growth, proliferation, metastasis, and epithelial‐mesenchymal transition [25]. Induction of ferroptosis has been demonstrated to sensitize tumor cells to radiotherapy [37–39]. In glioma therapy, ferroptosis‐inducing agents not only directly kill tumors but also enhance tumor sensitivity to radiochemotherapy [40]. Unfortunately, cancer cells can reduce the efficacy of cancer treatment by negatively regulating iron handling. Our findings showed that Fer‐1 significantly mitigated the radiosensitization induced by CCND1 knockdown. Lowering mitochondrial Fe^2+^ levels inhibits ferroptosis, thereby promoting radiation resistance [7]. In this study, we found that CCND1 knockdown significantly elevated intracellular ROS levels and lipid peroxidation, along with increased Fe^2+^ levels and decreased GSH content, thereby impacting cancer cell susceptibility to ferroptosis. Importantly, all these changes were reversed by the ferroptosis inhibitor Fer‐1. These findings suggest that CCND1 affected tumor sensitivity to radiotherapy through the regulation of ferroptosis. Of note, we also observed signs of pyroptosis upon CCND1 knockdown, suggesting that multiple cell death pathways may contribute to the radiosensitization, albeit with ferroptosis playing the predominant role.

Recent research has highlighted the increasing application of the ubiquitin‐proteasome system in cancer, where ubiquitin‐mediated protein degradation plays a pivotal role in diverse cellular processes linked to tumorigenesis [41, 42]. Deubiquitination is a reversible process that removes ubiquitin tags from proteins, thereby counteracting ubiquitin‐dependent degradation and maintaining the dynamic stability of protein levels [43]. USP7 is a highly abundant deubiquitinase that plays key roles in cell cycle progression, apoptosis, and DNA damage repair [44, 45]. Moreover, USP7 stabilizes various oncoproteins through its deubiquitinating activity, promoting malignant progression in cancer [46, 47]. In this study, we found that USP7 specifically mediates K48‐linked deubiquitination of CCND1, thereby preserving its stability in glioblastoma cells. Furthermore, overexpression of USP7 significantly attenuated the radiosensitivity and ferroptosis induced by CCND1 silencing and also reversed the effects of CCND1 knockdown on cell proliferation and apoptosis. These findings underscore the importance of the USP7‐CCND1 axis as a potential therapeutic target in glioblastoma.

Several limitations of this study should be acknowledged. First, multicenter case collection and expansion of sample sizes are needed to further corroborate our findings. Furthermore, although we confirmed the involvement of pyroptosis, the relative contribution of ferroptosis versus pyroptosis to CCND1 depletion–mediated radiosensitization remains to be fully elucidated. Future studies using genetic ablation of key executioners of each pathway are needed to determine the predominant mechanism.

In conclusion, our study identified CCND1 as a crucial prognostic biomarker in glioma, with high expression linked to poor outcomes. CCND1 knockdown impaired proliferation and promoted apoptosis while enhancing radiosensitivity through ferroptosis. Additionally, USP7 stabilized CCND1 via K48‐linked deubiquitination, thereby counteracting the effects of CCND1 silencing and restoring cell viability. These findings underscore the potential of targeting the USP7‐CCND1 axis to improve radiotherapy efficacy and treatment outcomes in glioma patients.

## Author Contributions

Yayu Zhuo, Chenrui Zhang, and Jianping Sun conceived this study. Yayu Zhuo, Chenrui Zhang, Xiaoyu Li, Xiaoyu Gu, Feng Wang, and Zishuo Du carried out experiments. Yiren Rong, Di Zhang, Haie Han, and Haijuan Mi analyzed the data. Yayu Zhuo and Jianliang Wu prepared the first draft of this manuscript. Jianping Sun revised this manuscript critically.

## Funding

This article has received support from Hebei Natural Science Foundation (H202520660) and Hebei Provincial Health Commission Medical Research Project (Grant 20211355).

## Disclosure

All authors have read and approved the final manuscript.

## Ethics Statement

This study does not involve human and animal experiments.

## Conflicts of Interest

The authors declare no conflicts of interest.

## Supporting Information

Additional supporting information can be found online in the Supporting Information section.

## Supporting information


**Supporting Information 1** Figure S1: (A) LDH release was measured following CCND1 silencing and pyroptosis inhibitor (DSF and Z‐YVAD‐FMK) treatment.  ^∗∗∗^ *p* < 0.001 vs. sh‐NC; ^###^ *p* < 0.001 vs. sh‐CCND1. (B) Representative Western blot of GSDMD and GSDMD‐N in U251 and U87 cells treated with different groups. (C) Representative Western blot of pro‐caspase‐1, cleaved caspase‐1, IL‐18, and IL‐1β in U251 and U87 cells treated with different groups. (D) RT‐qPCR was used to verify the reduction of USP7 mRNA levels following shRNA‐induced knockdown in glioma cells.  ^∗∗∗^ *p* < 0.001 vs. sh‐NC. Experiments were performed in triplicate.

**Supporting Information 2** Figure S2: (A) Western blot and statistical analysis showing the prolonged half‐life of CCND1 in U251 cells transfected with oe‐NC or oe‐USP7 and treated with CHX for the indicated times. (B) Western blot analysis of CCND1 protein levels in sh‐NC and sh‐USP7 transfected U251 cells treated with or without the proteasome inhibitor MG132 (10 μM) for 6 h. (B) RT‐qPCR was performed to assess the overexpression of USP7 in U251 and U87 cells.  ^∗∗∗^ *p* < 0.001 vs. oe‐NC. (C) USP7 protein levels were quantified to validate successful overexpression by Western blot.

## Data Availability

The data that support the findings of this study are available from the corresponding author upon reasonable request.
